# Research on the combination of color channels in heart rate measurement based on photoplethysmography imaging

**DOI:** 10.1117/1.JBO.26.2.025003

**Published:** 2021-02-23

**Authors:** JongSong Ryu, SunChol Hong, Shili Liang, SinIl Pak, Qingyue Chen, Shifeng Yan

**Affiliations:** a Northeast Normal University, School of Physics, Changchun, Jilin, China; b University of Science, Faculty of Physics, Pyongyang, Democratic People’s Republic of Korea; c Academy of Ultramodern Science, Kim Il Sung University, Pyongyang, Democratic People’s Republic of Korea; d Kim Chaek University of Technology, Faculty of Communication, Pyongyang, Democratic People’s Republic of Korea

**Keywords:** combination of color channels, heart rate, photoplethysmography imaging, plane-orthogonal-to-skin based method, prior-knowledge-based method

## Abstract

**Significance:** The measurement of human vital signs based on photoplethysmography imaging (PPGI) can be severely affected by the interference of various factors in the measurement process; therefore, a lot of complex signal processing techniques are used to remove the influence of the interference.

**Aim:** We comprehensively analyze several methods for color channel combination in the color spaces currently used in PPGI and determine the combination method that can improve the quality of the pulse signal, which results in a modified plane-orthogonal-to-skin based method (POS).

**Approach:** Based on the analysis of the previous studies, 13 methods for color channel combination in the different color spaces, which can be seen as having potential abilities in measuring vital signs, were compared by employing the average value of signal-to-noise ratio (SNR) and the box-plot in the public databases UBFC-RPPG and PURE. In addition, the pulse signal was extracted through the dual-color space transformation (sRGB → intensity normalized RGB → YCbCr) and fine-tuning on the CbCr plane.

**Results:** Among the 13 methods for color channel combination, the signal extracted by the Cb+Cr combination in the YCbCr color space includes the most pulse information. Furthermore, the average SNR of the modified POS for all the used databases is improved by 69.3% compared to POS.

**Conclusions:** The methods using prior knowledge are not only simple to calculate but can significantly increase the SNR, which will provide a great help in the practical use of vital sign measurements based on PPGI.

## 1 Introduction

Photoplethysmography imaging (PPGI) is a non-invasive and non-contact technology that can measure and monitor human vital signs such as the heart rate (HR), respiratory rate (RR), heart rate variability (HRV), and blood oxygen saturation (SpO_2_) by measuring the minute changes in the skin color due to the heartbeat with a normal camera.^1^ PPGI technology has been attracting a lot of attention from researchers because of its several advantages. First, it does not cause any discomfort to the people and also not interfere with their activities. Next, it allows measurement of human cardiac activities with only a smartphone, which requires a low cost to implement. The amount of hemoglobin changes in the microvascular network in the dermis due to the systole and diastole of the heart. At the same time, the absorption of light also changes according to the amount of hemoglobin, and it makes a variation in the intensity of the reflected light. Based on this fact, PPGI technology extracts the pulse signal from the image of the exposed skin. Therefore, PPGI technology requires not only sufficient knowledge of the light reflection mechanism on the skin and the transformation relationship between the color spaces but also the various signal processing techniques.

In Refs. 2–5, the PPGI-based HR measurement techniques studied in recent years have been outlined and systematically described by dividing them into the various categories from the different perspectives. Most of the techniques for measuring vital signs by PPGI can be seen as the signal extraction techniques by the linear and nonlinear combination of the color channel signals. In other words, only the method of obtaining the combination coefficients of the color channel signals is different. The methods by combining the R, G, and B channels can be classified into two categories: (1) blind source separation (BSS)-based method and (2) prior knowledge-based method.

Since Ref. 6 applied the independent component analysis (ICA) to the PPGI-based HR measurement, many methods based on BSS have been employed.^7^^–^^14^ However, the BSS-based methods have some limitations such as the order uncertainty, the requirement for the signal length, and the computational complexity; therefore, a series of problems can be raised in ensuring the automatism and real-time performance.

The methods based on prior knowledge can be divided into the following four classes. The first class is one that uses the individual color channels in the different color spaces. The sRGB color space is based on three basic colors including red, green, and blue, and through the different combinations of these three colors, any color in nature can be synthesized. The sRGB color space is commonly used in computer systems, cameras, and videos, and thus, it is the most widely employed in HR measurement based on PPGI. Reference 15 revealed that in the sRGB color space, the G channel had the most pulse information, and the R and B channels also had a certain amount of the pulse information. After that, several studies^16^^,^^17^ measured HR using the G channel in the sRGB color space. In Ref. 18, a study on the effectiveness of YUV color space in HR measurement based on PPGI was conducted. The YUV color space consists of a luma component (Y) and two chrominance components (U: B-Y, V: R-Y), and it is used as a default for the Android OS. By using the Android SDK, the captured frames can be stored in a data buffer with YUV format, so it can be considered as being practically more efficient in processing YUV data than RGB data. Comparing the channel signals between the sRGB and the YUV color spaces, it was found that using the V channel of the YUV color space had a better performance than either using all the channels of the sRGB color space or applying ICA or PCA to all the channels of the sRGB color space.^18^ In the HR measurement based on PPGI, the CIE Lab color space is also used. It is composed of a lightness component (L) and two chromatic components (a* and b*), in which the Euclidean distance between two different colors is similar to the color difference perceived by the human eye.^19^ In Ref. 20, the channels in the sRGB and the CIE Lab color spaces were compared and analyzed. As a result, it was proved that a* channel performed better than b* channel. In addition, it was shown that the signal of the a* channel had more pulse information than the G channel of the sRGB color space. In Ref. 21, comparisons of the R, G, B, and hue color channels were conducted. As a result, the hue channel showed the best performance among the four color channels. Meanwhile, in Ref. 22, the channel signals for the seven color spaces (sRGB, HSL, HSV, HIS, XYZ, CIE XYZ, and CIE YUV) were compared, and their performances were compared with ICA. The result verified that using the hue channel of HSV/HSL/HIS could measure HR more accurately than using all channels of the seven color spaces or ICA. HSL/HSV/HSI color spaces are cylindrical-coordinate color systems, where H is hue, S means saturation, L stands for lightness, V denotes value, and I represents intensity. Also, in all three color spaces, the hue channel is the same and the saturation channel is different. Variations in the sRGB color space depend on the color of the object as well as on the intensity of the reflected light from the surface, whereas hue channel does not depend on lightness.^22^ These characteristics show that hue channel is suitable for HR measurement. The second class includes the methods using the chrominance. In Ref. 23, a study on HR measurement using YCbCr color space was performed. The YCbCr color space consists of a luminance component (Y) and two chroma components [the blue-difference chroma component (Cb) and red-difference chroma component (Cr)], which separates the intensity and chroma information from the color information. Based on this, it was shown that Cb and Cr channels in the YCbCr color space contained a certain amount of pulse information and these two channels had the anti-phase nature, which resulted in a good SNR.^23^ In addition, it was suggested that the quality of the signal could be improved by the chrominance in the RGB color space, of which G-B had the best SNR, and this chrominance signal was also used in Ref. 24. Moreover, in some papers,^25^ HR was measured by using the $-{\text{R}}_{n}+2\cdot {\text{G}}_{n}-{\text{B}}_{n}$, which was the intersection of the projection planes used in Refs. 24 and 26. The third one is based on the model. Model-based methods include the chrominance-based method (CHROM),^26^ the blood volume pulse signature method (PBV),^27^ and the plane-orthogonal-to-skin based method (POS).^24^ According to the experimental results of Ref. 24, the overall performance of POS was better than the G,^15^ G-R,^28^ ICA,^7^ PCA,^8^ CHROM, PBV, and 2SR.^29^ A detailed explanation of the model-based methods can be found in Ref. 24. The other methods except for three methods mentioned above belong to the fourth class. In Ref. 30, the G channel in the intensity normalized RGB color space was used, whereas in Ref. 31, the change of blood concentration due to arterial pulsation as a pixel quotient in log space was defined and it was employed to improve the quality of the signal. Obviously, prior knowledge-based methods extract the pulse signals by considering the prior knowledge of the color vectors, thus the computational complexity of the algorithm can be reduced, and it can be considered that the extracted signals contain the enough pulse information. However, most studies for measuring the vital signs did not perform the comprehensive analysis on the color channel combination of the color spaces, but selected a color space based on the analysis of the relative merits for the color channel combination in two or more color spaces, in which the process for HR estimation was performed.

In this paper, we first use the public database UBFC-RPPG and PURE to compare and analyze 13 color channel combinations that can be considered to have the potential abilities for the artifact reduction, among the color channel combinations in the different color spaces. As a result, for the two databases, it is verified that the quality of the pulse signal can be significantly improved by the simple color space transformation from the sRGB space to the intensity normalized RGB space and the Cb+Cr combination in the YCbCr color space contains the most pulse information. In addition, it is also revealed that the plane created by the two chroma channel Cb and Cr in the YCbCr color space is orthogonal to the skin-tone direction [***1*** = (1, 1, 1) in the temporally normalized RGB space], which has the same characteristics as the POS plane suggested in Ref. 12. Considering the results mentioned above, this paper proposes a modified POS to extract the pulse signals by projecting the intensity normalized RGB signals onto the CbCr plane and the fine-tuning on the CbCr plane. With the experimental results of Ref. 24, we conducted the performance comparison between the proposed method and POS by using two public databases, UBFC-RPPG^32^ and PURE.^30^

## 2 Materials and Methods

### 2.1 Materials

In this paper, the public databases UBFC-RPPG and PURE were used to conduct a comprehensive comparison of the methods for the color channel combination in the different color spaces and to make an examination of the performance for the proposed method.

#### 2.1.1 Public database UBFC-RPPG

Public database UBFC-RPPG provides 50 video data collected using Webcam (Logitech C920 HD pro) and the PPG (pulse rate and SpO2) information measured by the transmissive pulse oximeter CMS50E. The distance between the participant and the camera was about 1 m, and the videos were recorded with a frame rate of 30 fps and a resolution of 640×480. Data collection was performed in the different places, and the ambient light was used as a source of illumination. The participants’ skin colors are different, and among them there were some bearded men and also some people wearing glasses. This database consists of two datasets. Eight videos are included in the first dataset. During the recording, the participants maintained a static state, one of whom performed an exercise before the measurement experiment, whereas the other participants did not perform an exercise. The second dataset contains 42 videos. In this dataset, participants were supposed to play the mathematical games during the measurement in order to simulate the realistic situations such as the interaction between human and computer.

#### 2.1.2 Public database PURE

Public database PURE provides 60 videos recorded with a frame rate of 30 fps and a resolution of 640×480 using a camera at a distance of 1.1 m from the participants, and the PPG information measured using a finger clip pulse oximeter (pulox CMS50E). During the recording, the ambient light was used as the source of illumination. Ten participants participated in the data collection, of which there were 8 males and 2 females. Data collectors set up six different states [steady, talking, slow translation, fast translation, small rotation (about 20 deg), and medium rotation (about 35 deg)] before the experiment to study the effects of the various rigid and non-rigid motions on PPGI. All the participants were supposed to maintain each state for 1 min.

### 2.2 Extracting Raw Signal in sRGB Color Space from the Video Data

The process for extracting the raw signal of the sRGB color space from the video data can be divided into two steps: (1) detection and tracking of the region of interest (ROI) and (2) spatial averaging.

**Fig. 1:**
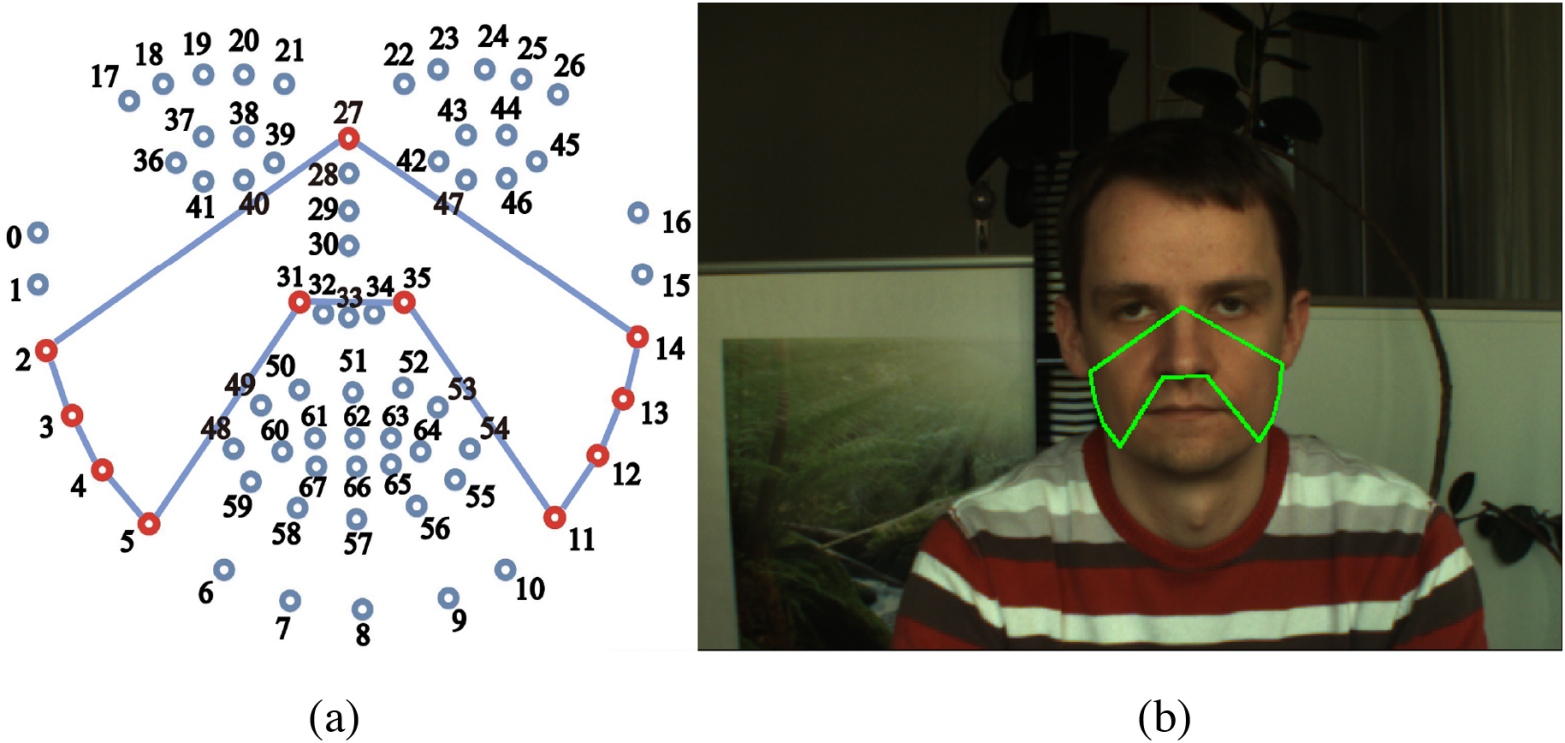
Selection of the facial ROI: (a) arrangement of 68 facial landmarks and (b) participant’s facial ROI.

First, the subject’s front face was detected using a detector^33^ that combines the modified linear support vector machine and the histograms of oriented gradient, after which the predictive model (http://dlib.net/files/shape_predictor_68_face_landmarks.dat.bz2) that provided 68 facial landmarks was applied to the detected front face to obtain the (x,y) coordinates of the subject’s facial landmarks in the different postures. In this paper, considering the fact that forehead and cheeks in the exposed facial skin have more pulse information,^17^ we selected both cheek areas enclosed by 2, 3, 4, 5, 31, 35, 11, 12, 13, 14, and 27 among the estimated 68 facial landmarks as the ROI (see Fig. 1).

Second, the raw RGB signals $C_{\mathrm{PPGI}}^{i}(t)$ were obtained by performing the spatial averaging for each fame through the individual R, G, B channels of the selected ROI and concatenating them according to the frame order.

$$C_{\mathrm{PPGI}}^{i}(t)=\frac{\underset{x,y\in \Omega_{\mathrm{fROI}}}{\sum }C^{i}(x,y,t)}{\left|\Omega_{\mathrm{fROI}}\right|},i\in \{R,G,B\},$$ (1)

where $C^{i}(x,y,t)$ is the pixel value at the coordinates (x,y) in the i channel at the time t and $\left|\Omega_{\mathrm{fROI}}\right|$ represents the area of the facial ROI. Like other sensors, the sensors for the sRGB color camera also cause the quantization noise. If a sufficient amount of the sensors for the RGB camera contributed to obtaining a facial ROI image, this quantization noise can be neglected by the spatial averaging in Eq. (1).

### 2.3 Different Combinations of R, G, and B Channels

It is possible to transform the sRGB color space and the other color spaces to each other; therefore, the combination of the color channels in the different color spaces can be seen as the combination of the R, G, B channels in the sRGB color space. In this paper, based on the analysis of Sec. 1, we compared the R, G, B channels of the sRGB color space, the chrominances $P\_C=2G_{n}-R_{n}-B_{n}$, $G\_B=G_{n}-B_{n}$ in the temporally normalized RGB color space, the G channel in the intensity normalized RGB color space, the a*, b* channels in the CIE Lab color space, the hue channel in HSI color space, the Cb, Cr channels and the Cb+Cr combination in the YCbCr color space, and the pixel quotient.^31^

The sRGB color space is commonly used in computers, TVs, and videos. In general, images in sRGB color space can be easily obtained in everyday life. The temporally normalized RGB color space can be represented by Eq. (2), whereas the chrominance signals in the temporally normalized RGB color space can be written by Eqs. (3) and (4). On the other hand, the intensity normalized RGB color space and the signal obtained by using the pixel quotient can be described as Eqs. (5) and (6), respectively.

$$X_{n}^{i}(t)=\frac{C_{\mathrm{PPGI}}^{i}(t)}{\mu (C_{\mathrm{PPGI}}^{i}(t))},i\in \{R,G,B\},$$ (2)

$$G\_B=X_{n}^{G}(t)-X_{n}^{B}(t),$$ (3)

$$P\_C=-X_{n}^{R}(t)+2\cdot X_{n}^{G}(t)-X_{n}^{B}(t),$$ (4)

$$Y_{n}^{i}(t)=\frac{C_{\mathrm{PPGI}}^{i}(t)}{\underset{j\in \{R,G,B\}}{\sum }C_{\mathrm{PPGI}}^{j}(t)},i\in \{R,G,B\},$$ (5)

$$Q(t)=\log\frac{G(t+1)\cdot R(t)}{R(t+1)\cdot G(t)}.$$ (6)

In Eq. (2), $C_{\mathrm{PPGI}}^{i}(x,y,t)$ is the raw temporal signal obtained from the sRGB video by the method mentioned in Sec. 2.2, and μ(·) represents the average operator taking an average value.

The CIE Lab color space consists of a lightness component (L value, ranging from 0 to 100) and two chromatic components (ranging from −120 to +120) i.e., a* component (from green to red) and b* component (from blue to yellow). In the measurement of the vital signs by PPGI, the interference of the illumination variations or the motion artifacts can only affect the lightness of color rather than the chromaticity.^20^ In addition, the cardiac activities cause the chromaticity change of the skin, and for this reason, CIE Lab color space can be considered as suitable for the purpose of use in PPGI. The transformation from the sRGB color space to the CIE Lab color space can be carried out as in Ref. 34.

The HSI color space consists of the hue (range: 0 deg to 360 deg), the saturation (range: 0 to 1), and the intensity (range: 0 to 1). The signals of the intensity and the saturation channels can be affected by the illumination variation, which means that they are not suitable for HR measurement by PPGI. The transformation from the sRGB color space to the HSI color space can be implemented as in Ref. 21.

The YCbCr color space is one of the important color spaces that can separate the intensity from the color information. Here, Y is the luminance component, whereas Cb and Cr stand for the blue-difference and red-difference chroma components, respectively. If the scaling constant value can be ignored, the relationship between the RGB space and the YCbCr space is as follows:

$$(\begin{array}{l} Y\\ \mathrm{Cb}\\ \mathrm{Cr} \end{array})=(\begin{array}{ccc} 0.299 & 0.587 & 0.114\\ -0.168 & -0.331 & 0.499\\ 0.499 & -0.418 & -0.081 \end{array})\cdot (\begin{array}{l} R\\ G\\ B \end{array}).$$ (7)

### 2.4 Pulse Extraction by Modified POS

#### 2.4.1 POS

First, recall the conventional POS, which consists of three steps, i.e., the temporal normalization, the projection, and the fine-tuning. The temporal normalization can be performed by Eq. (2). The projection step of projecting the temporally normalized RGB signals $X_{n}^{i}(t)=({\text{r}}_{n}(t),{\text{g}}_{n}(t),{\text{b}}_{n}(t){)}^{T}\in R^{3\times N}$ (N is the total number of frames) onto the POS plane (Eq. (8) and the fine-tuning (i.e., α-tuning) step (Eq. (9) are the most important steps of POS.

$$S(t)=U\cdot X_{n}^{i}(t)\;s.t\{\begin{array}{l} U\cdot 1={\left(0,0\right)}^{T}\\ u_{m}^{T}\cdot u_{p}=0 \end{array},$$ (8)

$$p(t)=s_{p}(t)+\alpha s_{m}(t)\;\text{with }\alpha =\frac{\sigma (s_{p}(t))}{\sigma (s_{m}(t))}.$$ (9)

In Eq. (8), $1={\left(1,1,1\right)}^{T}$, and $U=(u_{p},u_{m}{)}^{T}\in R^{2\times 3}$ is a projection matrix, which satisfies the constraint of Eq. (8). In POS, the two projection axes are set as $u_{p}={\left(0,1,-1\right)}^{T}$, $u_{m}={\left(-2,1,1\right)}^{T}$. Also, $S(t)=(s_{p}(t),s_{m}(t){)}^{T}\in R^{2\times N}$ is the result of projecting $X_{n}^{i}(t)$ onto the POS projection axes. In Eq. (9), p(t) is the BVP signal obtained by the fine-tuning, and σ(·) means the standard deviation operator.

#### 2.4.2 Modified POS

As can be seen from Eq. (7), which shows the transformation between the sRGB and the YCbCr color spaces, two Cb and Cr color vectors are orthogonal to the normalized skin-tone direction. In other words, the Cb and Cr color vectors in the YCbCr color space are placed on the POS plane and they satisfy Eq. (10),

$$\left\{ \begin{array}{l} {\left(u_{\mathrm{Cb}},u_{\mathrm{Cr}}\right)}^{T}\cdot 1=(0,0{)}^{T}\\ u_{\mathrm{Cb}}^{T}\cdot u_{\mathrm{Cr}}≍0 \end{array}.\right.$$ (10)

In addition, the signals of the Cb and Cr channels contain a certain amount of the BVP signal, and the signals of these two channels have the anti-phase nature. This anti-phase nature and Eq. (10) suggest that the Cb and Cr color vectors can replace the original projection axes of POS [i.e.,$u_{p}={\left(0,1,-1\right)}^{T}$, $u_{m}={\left(-2,1,1\right)}^{T}$]. Moreover, according to the analysis result of Ref. 30, it can be seen that the signal quality can be improved by the intensity normalization (simple scaling for sRGB space). Based on this fact, in this paper, first, the raw temporal color signals in the sRGB color space are intensity normalized, and then the pulse signal is extracted by applying the modified POS in which the projection matrix U in Eq. (8) is replaced with $U_{\mathrm{CbCr}}$.

$$U_{\mathrm{CbCr}}={\left(u_{\mathrm{Cb}},u_{\mathrm{Cr}}\right)}^{T}=(\begin{array}{ccc} -0.168 & -0.331 & 0.499\\ 0.499 & -0.418 & -0.081 \end{array}).$$ (11)

## 3 Results

In this paper, the public databases UBFC-RPPG and PURE were used to compare the combination methods mentioned in Sec. 2.3. First, the raw RGB signals were extracted by the method mentioned in Sec. 2.2, then the signals in the different color spaces were obtained by using the combination methods mentioned in Sec. 2.3, after which the SNRs of the signals were calculated by the method mentioned in Ref. 26. Finally, the performance comparison of the combination methods was conducted by using the average SNR and the box-plot of SNR. In the same way, POS and modified POS were compared. The equation for calculating the SNR of the signal described in Ref. 26 is as follows:

SNR=10log10(∑f=0.74(Ut(f)S^(t)2)∑f=0.74(1−Ut(f))S^(t)2), (12)

where S(f) represents the spectrum of the pulse signal (f is the frequency) within 0.7 and 4 Hz, and U(f) indicates a binary template window, which has two values 1 and 0 (1: the case within two frequency windows, one of whom is near the fundamental frequency ($f_{\mathrm{HR}}$) [$f_{\mathrm{HR}}-0.1$, $f_{\mathrm{HR}}+0.1$], and the other is near the first harmonics [$2f_{\mathrm{HR}}-0.2$, $2f_{\mathrm{HR}}+0.2$], and 0: the case outside of the two frequency windows).

### 3.1 Comparison of Combination Methods

**Fig. 2:**
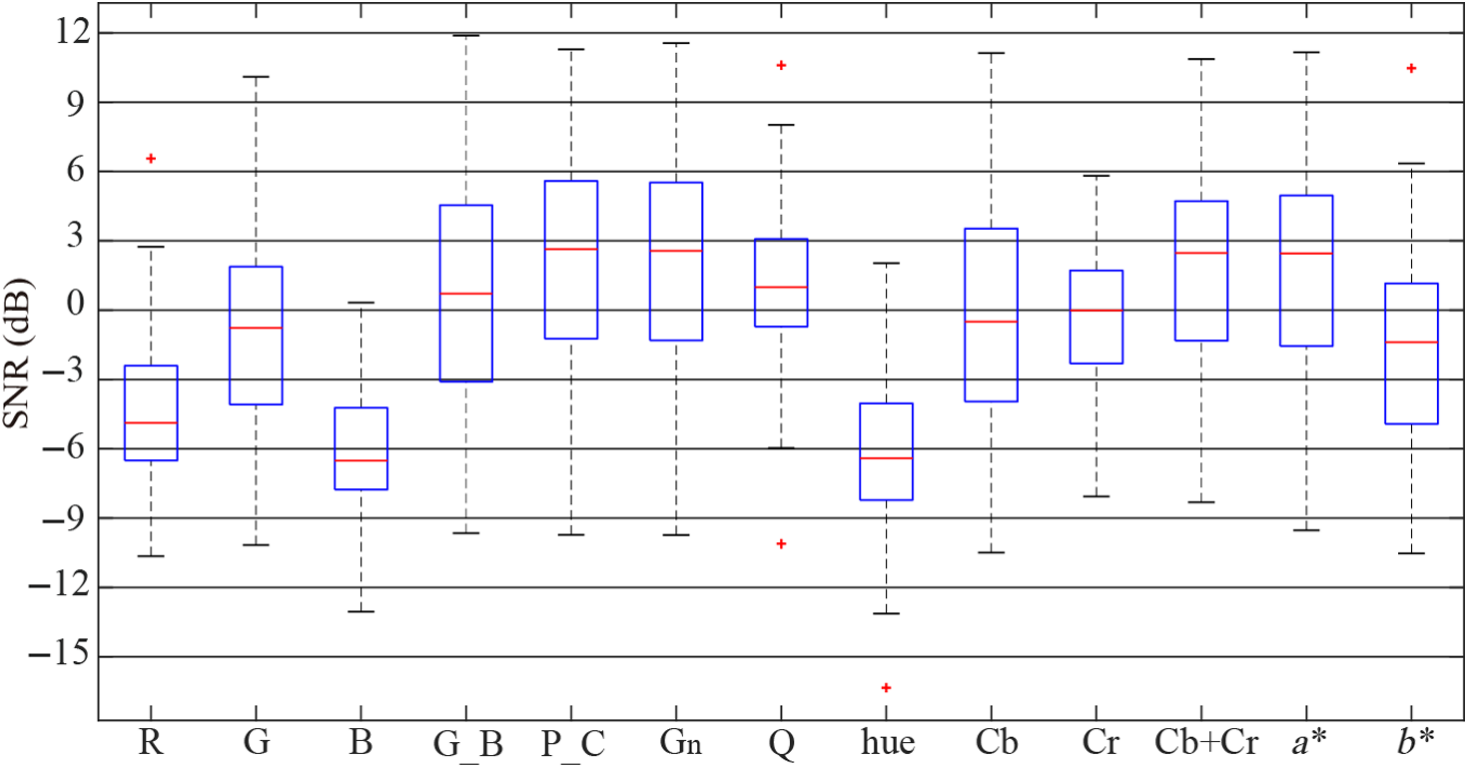
SNR comparison of the color channel combinations for the public database UBFC-RPPG.

**Fig. 3:**
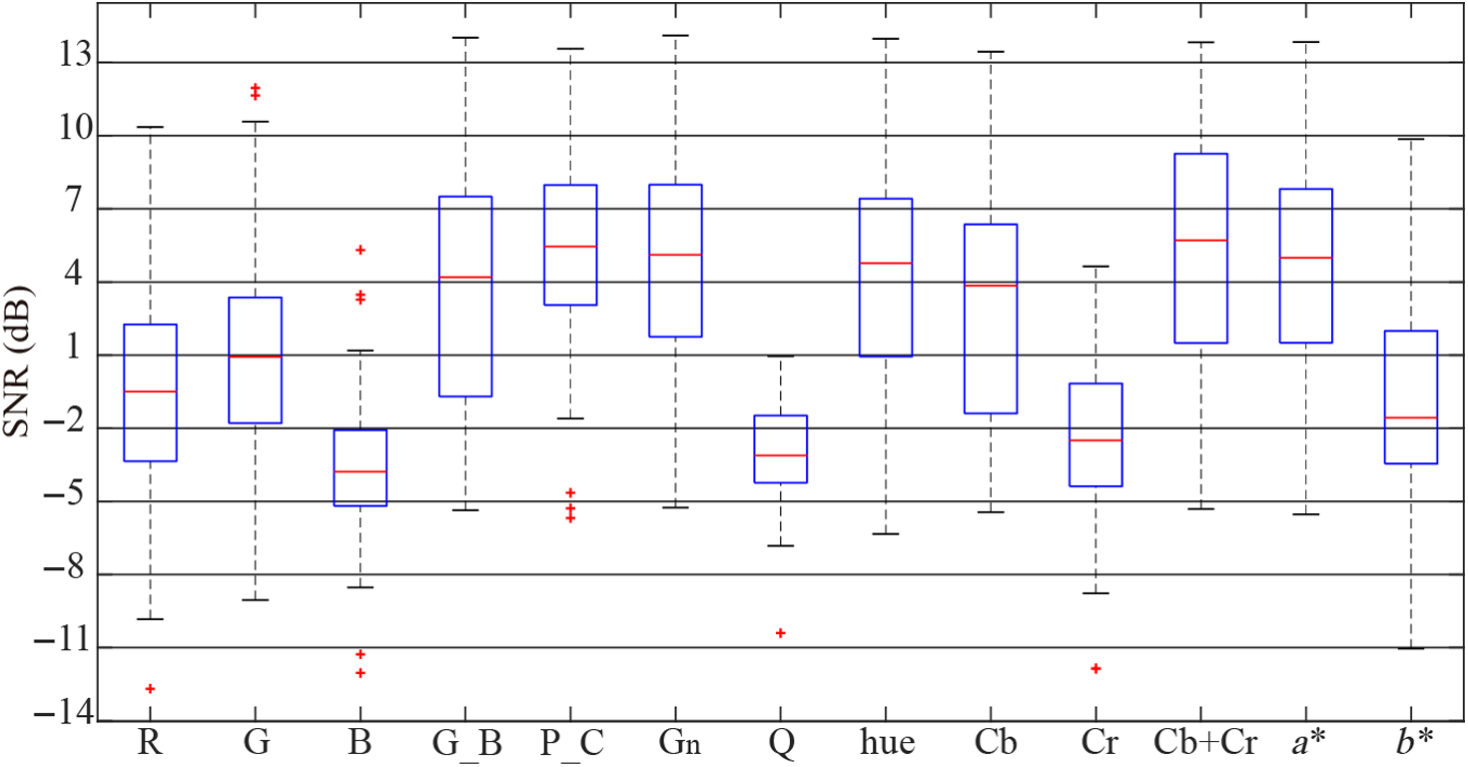
SNR comparison of the color channel combinations for the public database PURE.

Figures 2 and 3 show the box-plots of the SNRs for the pulse signals obtained by each combination method mentioned in Sec. 2.3, for the public databases UBFC-RPPG and PURE, and Table 1 lists their average SNRs. As can be seen in Table 1 and Fig. 2, for the public database UBFC-RPPG, the combination of Cb and Cr in the YCbCr color space (i.e., Cb+Cr), P_C combination in the temporally normalized RGB color space, G channel ($G_{n}$) in the intensity normalized RGB color space have better SNR than the other combinations, where their average SNRs are 2.10, 2.09, and 1.99, respectively. Meanwhile, from Table 1 and Fig. 3 for the public database PURE, it can be noted that the values of average SNRs for the signals obtained by Cb+Cr combination in the YCbCr color space and P_C combination in the temporally normalized RGB color space are 5.41 and 5.02, respectively, which indicates that they have better SNRs than the others.

**Table 1:** Comparison of the average SNRs for the color channel combinations.

	R	G	B	G_B	P_C	$G_{n}$	Q	hue	Cb	Cr	Cb+Cr	a*	b*
UBFC-RPPG	−4.27	−0.78	−6.18	0.72	2.09	1.99	0.84	−5.99	−0.51	−0.41	2.10	1.63	−1.39
PURE	−0.57	1.00	−3.68	4.00	5.02	4.79	−3.12	4.23	3.01	−2.34	5.41	4.59	−0.60

### 3.2 Comparison of POS and Modified POS

**Fig. 4:**
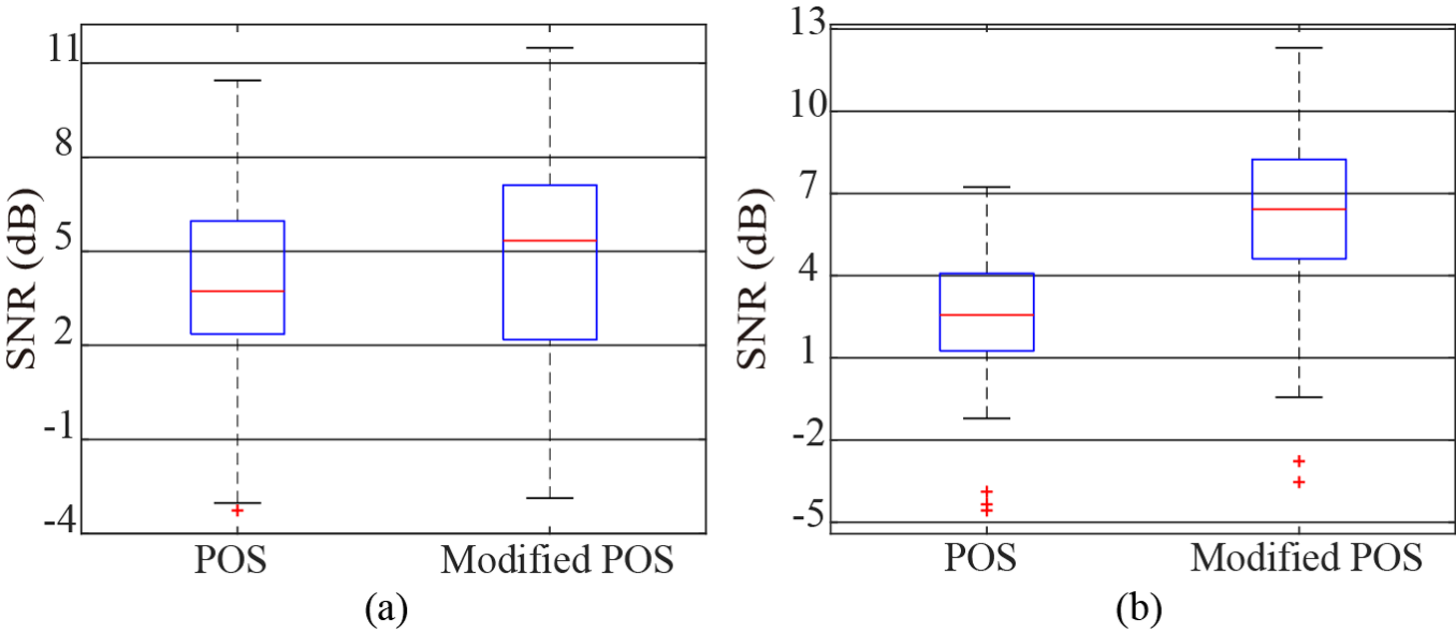
SNR comparison of POS and modified POS: (a) UBFC-RPPG and (b) PURE.

Figure 4 depicts the box-plots of the SNRs for the pulse signals obtained by POS and modified POS for the public databases UBFC-RPPG and PURE, and Table 2 lists their average SNRs. As can be shown in Table 2 and Fig. 4, the average SNRs of the modified POS for the two public databases UBFC-RPPG and PURE are 4.61 and 6.18, respectively, and the average SNRs of POS for the same two public databases are 3.54 and 2.51, respectively, which points out that the performance of the modified POS is better than that of POS.

**Table 2:** Comparison of the average SNRs for POS and modified POS.

	POS	Modified POS
UBFC-RPPG	3.54	4.61
PURE	2.51	6.18
Overall	3.03	5.13

Overall: mean value of the average SNRs of the two public databases for the compared methods.

**Fig. 5:**
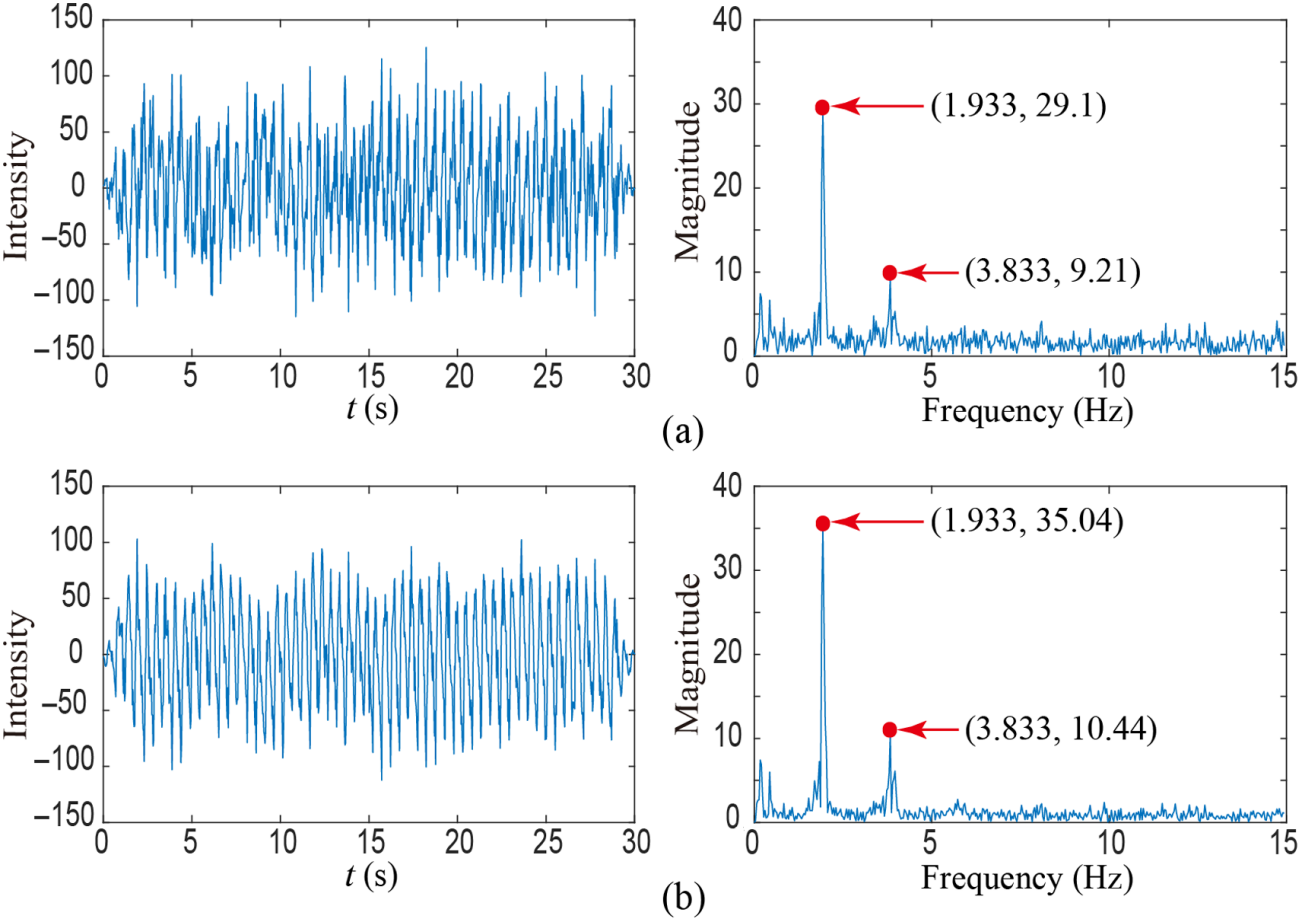
Comparison of the pulse signals extracted by POS and modified POS in the time and frequency domains: (a) POS and (b) modified POS.

In Fig. 5, the signals extracted by applying POS and modified POS to the data of the 35th participant in the public database UBFC-RPPG are plotted in the time and frequency domains. As can be seen in Fig. 5, it is obvious that the performance of the modified POS is better than that of POS.

## 4 Discussion

In this section, we comparatively and comprehensively analyze the methods of combining the color channels in the different color spaces and the proposed method through the results in Sec. 3. We also describe our opinions, the limitations of the proposed method and the future research directions.

In this paper, research was conducted in two areas. First, using two public databases, UBFC-RPPG and PURE, the effects of the color spaces and the color channel combinations on the measurement of the vital signs were analyzed through a comparative experiment of 13 single color channels and color channel combinations showing the relatively good HR estimation performances. As can be seen in Table 1, Figs. 2 and 3, the average SNRs of the G channel in the sRGB color space were −0.78 and 1.0, which indicated that G channel contained the most pulse information, followed by R and B. This result is the same as Ref. 15. The signals obtained by the chrominances (i.e., G_B and P_C) in the temporally normalized RGB color space had better SNRs than all channels in the sRGB color space. In particular, the average SNRs of the pulse signals extracted by the P_C combination were 2.09 and 5.02, which were significantly more improved than the G channel of the sRGB color space. In fact, in the temporally normalized RGB color space, the P_C color vector was one that lies in the intersection between the POS plane and the CHROM plane, so the P_C combination could reduce the artifacts to a certain degree. In addition, in the intensity normalized RGB color space, the average SNRs of the G channel signals were 1.99 and 4.79, which were significantly improved compared to the R, G, and B channels of the sRGB color space. This is because intensity normalization reduces the effect of global illumination changes and increases the effect of color hue.^30^ The signal obtained by the pixel quotient was slightly improved with an average SNR of 0.84 in the public database UBFC-RPPG, but on the contrary, the quality was deteriorated to −3.12 in the public database PURE. This implies that the performance of this method depends on the measurement conditions such as environment, camera, etc. In the YCbCr color space, a certain amount of the pulse information was included in the Cb and Cr channels. Furthermore, the average SNRs of Cb+Cr were 2.1 and 5.41, which could be considered that the quality of the signal was significantly improved. This is related to the fact that the pulse information is included in both the Cb and Cr channels, and at the same time, the artifacts in both channels have the anti-phase nature. In the CIE Lab color space, the average SNRs for a* channel were 1.63 and 4.59, which meant that a* channel had more pulse information than b*. This is due to the fact that blue and yellow in b* have a small skin penetration depth, so the performance is lower than a*. This result is the same as Ref. 20. Comprehensive analysis of the color channels and the combination methods mentioned in this paper shows that for both of the public databases, the signal obtained by the Cb+Cr combination has the most pulse information, followed by P_C. The SNRs of $G_{n}$ and a* are also relatively good. On the other hand, the performance of the hue channel is relatively good with an average SNR of 4.23 for the public database PURE, but on the contrary, it is very poor with an average SNR of −5.99 for the public database UBFC-RPPG, which indicates that the hue’s performance varies depending on the measurement conditions. Second, the improvement for the quality of the pulse signal was analyzed based on the dual-color space transformation and the compensation of the two color vectors. Obviously, the SNR of the pulse signal was significantly improved through the transformation from the sRGB space to the intensity normalized RGB color space (i.e., scaling), and the Cb+Cr combination in the YCbCr color space had the best SNR among the 13 combination methods. Meanwhile, in the YCbCr color space, the CbCr plane is one orthogonal to the normalized skin tone direction, which has the same characteristics as the POS plane. In consideration of these characteristics, we proposed a modified POS that combines the dual-color space transformation (sRGB space -> intensity standardized RGB space -> YCbCr space) and the fine-tuning, and compared its performance with POS. As can be seen in Table 2 and Fig. 4, the average SNR of the modified POS for all used databases was 5.13, which was better than POS with the average SNR of 3.03. In particular, the signal quality was significantly improved in the database PURE considering the motion, which implies that the effect is more remarkable in the condition that the artifacts exist.

To fairly evaluate the proposed method and other previous methods, we only described the core algorithm for the HR measurement but did not discuss the pre-processing or post-processing algorithms. In addition, only two relatively representative public databases were used for the comparative experiments, but it would be better to use the databases considering the more complex motion artifacts, the various illumination intensities and the dynamic illumination variations with the several kinds of cameras so that the comparative analysis gets more universal results. However, as we know, there is no public database that takes into account all of these possible practical conditions, and we will proceed with the more detailed measurement experiments in the future. Also, since each color space has the different characteristics, vital signs will be estimated more accurately if data fusion technology is applied based on the concrete analysis of the different color spaces using the sufficient databases.

## 5 Conclusion

In this paper, the methods of combining the color channels based on the prior knowledge of the color vectors were comprehensively compared by using two public databases, UBFC and PURE, and a modified POS was proposed. The comparison results showed that a simple color space transformation or a color channel combination could significantly improve the signal quality. In addition, it was verified that the Cb and Cr channel in the YCbCr color space could replace the two projection axes of the conventional POS, and the average SNR of the modified POS for the all databases was 69.3% improved compared to POS. At the same time, unlike the methods that do not use prior knowledge about color channel vectors such as BSS, the proposed method has the advantage of the lower calculational complexity. These advantages of the proposed method will be of great help in the practical use of the PPGI-based measurement for vital signs.
